# The role of immunotherapy in advanced renal cell carcinoma

**DOI:** 10.1590/S1677-5538.IBJU.2020.0681

**Published:** 2021-01-20

**Authors:** Ercília Rita Mondlane, Pedro Abreu-Mendes, Diana Martins, Rui Cruz, Fernando Mendes

**Affiliations:** 1 ESTeSC Politécnico de Coimbra Coimbra Portugal Politécnico de Coimbra, ESTeSC, DFARM, ESTeSC, SM Bispo, Coimbra, Portugal.; 2 Centro Hospital Universitário de São João Serviço de Urologia Porto Portugal Serviço de Urologia, Centro Hospital Universitário de São João, Porto, Portugal.; 3 Universidade do Porto Faculdade de Medicina Porto Portugal Faculdade de Medicina Universidade do Porto, Porto, Portugal.; 4 ESTeSC Politécnico de Coimbra Coimbra Portugal Politécnico de Coimbra, ESTeSC, DCBL, SM Bispo, Coimbra, Portugal.; 5 Universidade de Coimbra Instituto de Investigação Clínica e Biomédica de Coimbra Coimbra Portugal Universidade de Coimbra, Instituto de Investigação Clínica e Biomédica de Coimbra Coimbra, Portugal.; 6 Universidade de Coimbra Centro de Biomedicina e Biotecnologia Inovadoras (CIBB) Coimbra Portugal Universidade de Coimbra, Centro de Biomedicina e Biotecnologia Inovadoras (CIBB), Coimbra, Portugal.; 7 Centro Académico Clínico de Coimbra Coimbra Portugal Centro Académico Clínico de Coimbra (CACC), Coimbra, Portugal.; 8 Universidade do Porto Instituto de Investigação e Inovação em Saúde Porto Portugal Instituto de Investigação e Inovação em Saúde, Universidade do Porto, Porto, Portugal.

## INTRODUCTION

Cancer has become increasingly common worldwide, being the second leading cause of death and an important barrier to increasing life expectancy in all countries in the XXI century (1). The reasons behind these statistic numbers are complex, but they are associated with aging, population growth and the increased prevalence of risk factors (1).

Kidney cancers are ranked 14th in the World among the ones with the highest incidence (1-3). The renal cell carcinoma (RCC) represents 80-85% of all kidney cancers, and it is the most common and the third most diagnosed urogenital malignancy (2). It occurs usually in the sixth and seventh decades and most commonly in men (4). The incidence varies globally, with the highest rates in developed countries such as North America and Europe and the lowest rates in Asia and Africa (3).

Due to the high incidence and mortality levels of RCC, it is important to find the most appropriate therapeutic strategies, and also to analyse the influence of risk factors. Age (over 85 years), gender (male), smoking habit, analgesics use, obesity, lack of physical activity, exposure to industrial or environmental agents and comorbidities such as hypertension, urinary stones, diabetes, liver and chronic kidney diseases, are known factors related to the incidence of RCC (5). Currently, most of the RCC cases have been diagnosed through computed tomography or abdominal ultrasonography, in asymptomatic subjects (2).

RCC is divided into multiple subtypes according to its histological characteristics. The most common subtype is clear cell renal cell carcinoma (ccRCC) (2, 6), responsible for approximately 80% of all cases of RCC. The other major subtypes include papillary (12%), chromophobe (4%), oncocytoma (4%) and collecting duct (<1%). Familial RCC is often seen in the context of an inherited syndrome, such as Von Hippel-Lindau (VHL) syndrome and Birt-Hogg-Dubé syndrome (4, 6).

RCC’s treatment can be conducted following two pathways, namely: local treatment with nephrectomy or other ablative strategies (in small masses and older patients), or through systemic therapy; based on the disease staging. In most cases of localized renal cancer, partial or total nephrectomy can be used to eradicate the disease (2). However, the post-operative recurrence rate can be of 20-40% in the first 5 years and 5-10% in late recurrence (4). In cases of recurrence and progression after initial surgical treatment during follow-up or in cases of advanced renal cell carcinoma (aRCC) at presentation, the best treatment is systemic. Based on a further classification of aRCC as favourable, intermediate or poor prognosis, based on predetermined scores (3), the best systemic therapy varies.

Immunotherapy represents a relatively recent therapeutic approach in cancer treatment. With several advances in the last decade, this particular form of treatment is already considered extremely important in different cancer types (melanomas, lung, head, neck, urethra and kidney cell cancer) (7). Immunotherapy consists of using and enhancement of the immune system itself, for the detection and elimination of cancer cells, generating a durable response and effective regression, in addition to preventing metastases (6, 8, 9). Immunotherapeutic strategies include the use of immune system modulators, monoclonal antibodies (MAb), vaccines and, more recently, immune checkpoint inhibitors (7, 9, 10). This study aims to perform a systematic review in the use of the immune system as a therapeutic strategy to treat aRCC as well as its impact on patient survival and quality of life.

## MATERIAL AND METHODS

The literature used in this review is available on the indexed search engine “Pubmed/Medline”. The selected key words were “immunotherapy”, “advanced renal cell carcinoma”, “immune checkpoints inhibitors”, “monoclonal antibodies”, according to Medical Subject Headings (Mesh). The inclusion and exclusion criteria were created to guarantee the relevance and validity of the information. Therefore, the inclusion criteria were scientific articles and clinical trials (humans) with a publication date equal to or less than 5 years, availability of free-full text. The authors excluded papers in which the title, abstract, and content were not relevant to this study.

**Figure 1:**
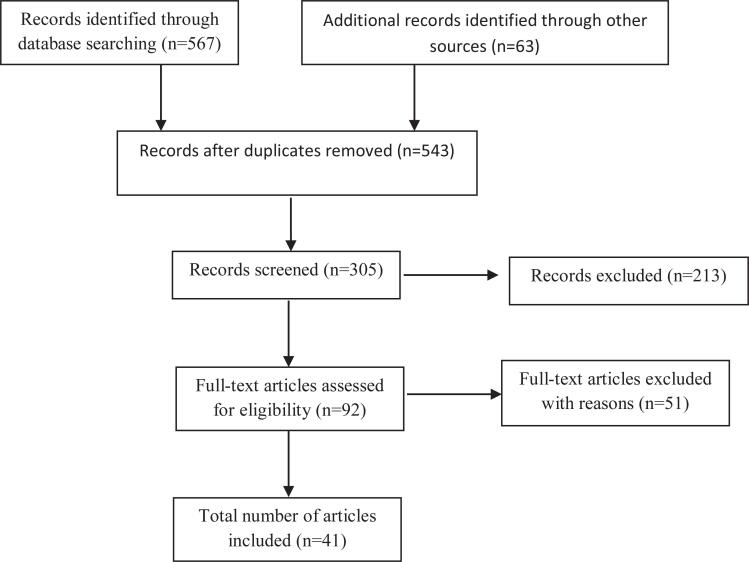
PRISMA flow diagram of study selection process.

The research strategies used are detailed in Figure-1, and all the sources that provided theoretical support were referenced (Figure-1).

## COMMENTS

### Cytokines

Cytokines were the first immunotherapeutic strategy to be used in clinical practice, with the approval of Interferon-α (IFN-α) in 1986. Injected cytokines directly stimulate the growth and activity of immune cells and there are 3 types of cytokines used in immunotherapy: IFN, interleukins (IL), and granulocyte-macrophage colony-stimulating factor (GM-CSF) (11).

### Interferon-α

IFN’s mechanism of action is based on the activation of T and natural killer (NK) cells and cell cycle inhibition (2). IFN-α is classified as a type I IFN and it comprises a family of more than 20 distinct variants, encoded by a cluster on chromosome 9. For all IFN-α subtypes action, a connection to a specific membrane receptor complex (IFN-AR) is necessary. This binding leads to the activation of intracellular signalling cascades that increase the expression and activation of signal transducers and transcription activators (STAT1, STAT2 and STAT3) (12). STAT1 is the most implicated in cell death programmed by IFN-α. IFN-AR are not only expressed in malignant cells, but also in non-neoplastic cells and it makes the risk of developing higher (12) adverse events (AE). A phase III study concluded that only a small number of patients experienced a complete response with IFN-α monotherapy and the AE related made it difficult to evaluate the long-term use (13). Results of the most recent study about the use of IFN-α in aRCC are presented in Table-1.

**Table 1:** Results of clinical trial articles (conducted between 2015 and 2020) included in the review.

Drug	Authors/Year	Trial	Results	AE observed (any grade)
INF-α	Eto et al. (2015) (43)	Phase II study evaluated the combination of IFN-α with sorafenib in 42 patients with confirmed aRCC.	- ORR was 26.1%; - Median OS was not reached; - Grade 3/4 AE were observed 42% of the patients discontinued treatment due to AE;	Hand foot skin reaction (64.3%); malaise (57.1%); rash (52.4%), diarrhoea (47.6%); thrombocytopenia (45.2%)
Interleukin-2	Donskov et al. (2018) (44)	Phase II study compared IL-2 plus IFN-α plus bevacizumab versus IL-2 plus IFN-α in 118 patients with favourable or intermediate risk.	- ORR was 44.1% (IL2+INF+BEV) versus 28.8% (IL2+INF). - Median OS was 30.3 months (IL2+INF+BEV) versus 34.1 months (IL2+INF); - Grade 3/4 AE occurred in 64% (IL2+INF+BEV) versus 61% (IL2+INF) of the patients	IL+INF+BEV: fatigue (97%); flu like symptoms (95%); nausea (90%); dry skin (71%); diarrhoea (64%); IL2+INF: fatigue (95%); flu like symptoms (93%); nausea (88%); dry skin (81%); diarrhoea (73%)
AGS-003	A. Amin et al. (2015) (22)	Phase II study evaluated the combination of sunitinib plus AGS-003 in 21 patients with intermediate or poor prognostic.	- No complete responses were observed 62% experienced a clinical benefit (42.9% correspond to partial response and 19.0% to stable disease); - Median OS was 30.2 months; - 42.9% experienced grade 3 AE associated with sunitinib. No grade 4 AE was reported	Diarrhoea (59%); fatigue (59%); nausea (55%); rash (46%); weight decrease (41%)
IMA901	Rini et al. (2016) (24)	Phase III study (Imprint) compared the clinical effect of IMA901 plus sunitinib versus sunitinib monotherapy in 139 patients.	- Median OS was 33.17 months (IMA901+SUN) versus not reached (SUN); - 57% of the patients (IMA901+SUN) versus 47% (SUN) experienced grade 3/4 AE.	* IMA901+SUN: hypothyroidism (27%); diarrhoea (26%); PPE syndrome (23%); fatigue (19%); nausea (19%). SUN: diarrhoea (26%); PPE syndrome (25%); hypothyroidism (23%); fatigue (19%); hypertension (18%);
Atezolizumab	Mcdermott et al. (2018) (62)	Phase II (IMmotion150) compared atezolizumab monotherapy, atezolizumab plus bevacizumab versus sunitinib in 305 patients in ITT and PD-L1+populations.	In the ITT population: - Median ORR was 32% (ATE+BEV) versus 29% (SUN) versus 25% (ATE); In the PD-L1+population: - Median ORR was 46% (ATE+BEV) versus 27% (SUN) versus 28% (ATE); - Median OS was not presented for both groups; - Grade 3/4 AE occurred in 40% (ATE+BEV) versus 57% (SUN) versus 17% (ATE).	Not referred.
	Rini et al. (2019) (61)	Phase III study (IMmotion151) compared the efficacy and safety of atezolizumab plus bevacizumab versus sunitinib in 915 patients in ITT and PD-L1+populations.	In the ITT population: - ORR was 37% (ATE+BEV) versus 33% (SUN); - Median OS was 33.6 months (ATE+BEV) versus 34.9 months (SUN). In the PD-L1+ population: - ORR was 43% (ATE+BEV) versus 35% (SUN); - Median OS was 34.0 months (ATE+BEV) versus 32.7 months (SUN); - Grade 3/4 AE occurred in 40% (ATE+BEV) versus 54% (SUN);	ATE+BEV: hypertension (33%); fatigue (28%); diarrhoea (20%); proteinuria (20%); asthenia (15%). SUN: diarrhoea (47%); PPE syndrome (43%); hypertension (40%); fatigue (33%); nausea (31%).
Avelumab	Choueiri et al. (2018) (58)	Phase IB study (JAVELIN Renal 100) evaluated the combination of avelumab plus axitinib as first-line treatment in 55 patients.	- ORR was 58%; - Grade 3/4 AE occurred in 58% of the patients.	Diarrhoea (58%); dysphonia (47%); hypertension (47%); fatigue (46%); palmar-plantar erythrodysesthesia syndrome (31%);
	Motzer RJ et al. (2019) (60)	Phase III study (JAVELIN Renal 101) compared the combination of avelumab plus axitinib versus sunitinib as first-line treatment, in 886 patients.	In the ITT population: - ORR was 51.4% (AVE+AXI) versus 25.7 (SUN). In the PD-L+ population: - ORR was 55.2% (AVE+AXI) versus 25.5% (SUN); - Grade 3/4 AE occurred in 71.2% (AVE+AXI)) versus 71.5% (SUN).	AVE+AXI: diarrhoea (62.2%); hypertension (49.5); fatigue (41.5%); nausea (34.1%); palmar-plantar erythrodysesthesia syndrome (33.4%).
				SUN: diarrhoea (47.6%); fatigue (40.1%); nausea (39.2%); hypertension (36.0%); PPE syndrome (33.7%).
	Vaishampayan et al. (2019) (57)	Phase IB study evaluated the use of avelumab monotherapy as first or second line treatment in 82 patients.	In the first-line treatment: - ORR was 16.1%; - Median OS was not reached. In the second-line treatment: - ORR was 10%; - Median OS was 16.9 months; - Grade 3/4 AE occurred in 12.9% (first-line) and 5.0% (second line).	In the first-line treatment: pruritus (19.4%); fatigue (17.7%); asthenia (14.5%); nausea (14.5%); pyrexia (12.9%). In the second-line treatment: infusion-related AE (30.0%); fatigue (25.0%); any immune-related AE (15.0%); diarrhoea (15.0%); pyrexia (10.0%).
Nivolumab	Motzer et al. (2015) (45)	Phase III study (Checkmate 025) compared nivolumab versus everolimus in 821 previously treated patients.	- ORR was 25% (NIV) versus 5% (EVE); - Median OS was 25.0 months (NIV) versus 19.6 months (EVE); -Grade 3/4 AE occurred in 19% of the patients (NIV) and 37% (EVE).	NIV: fatigue (33%); nausea (14%); pruritus (14%); diarrhoea (12%); decreased appetite (12%). EVE: fatigue (34%); stomatitis (30%); diarrhoea (21%); decreased appetite (21%); rash (20%).
	Amin et al. (2018) (49)	Phase I study (Checkmate 216) compared the safety and efficacy of nivolumab plus sunitinib versus nivolumab plus pazopanib in 53 patients.	- ORR was 55% (NIV+SUN) versus 45% (NIV+PAZ); - Median OS was not reached (NIV+SUN) versus 27.9 months (NIV+PAZ); - Grade 3/4 AE occurred in 81.8% (NIV+SUN) versus 70% (NIV+PAZ).	NIV+SUN: fatigue (84.8%); diarrhoea (63.6%); dysgeusia (63.6%); nausea (57.6%); hypertension (48.5%). NIV+PAZ: nausea (75.0%); fatigue (60.0%); diarrhoea (60.0%); dysgeusia (63.6%); decreased appetite (40.0%)
Pembrolizumab	Atkins et al. (2018) (54)	Phase IB study evaluated the combination of axitinib plus pembrolizumab in 52 patients.	- ORR was 73%; - Median OS was not reached, but at 18 months, the probability of being alive was 93.9%; - Grade 3/4 AE occurred in 65% of the patients.	* Fatigue (63%); diarrhoea (62%); dysphonia (46%); increased alanine aminotransferase concentration (29%); hypertension (27%)
	Rini et al. (2019) (53)	Phase III (Keynote-426) study compared the combination of pembrolizumab plus axitinib versus sunitinib in 861 treatment-naïve patients.	-ORR was 59.3% (PEM+AXI) versus 35.7% (SUN); -At 12 months, the percentage of patients alive was 89.9% (PEM+AXI) versus 78.3% (SUN); -Grade 3/4 AE occurred in 75.8% (PEM+AXI) versus 70.6% (SUN);	PEM+AXI: diarrhoea (54.3%); hypertension (44.5%); fatigue (38.5%); hypothyroidism (35.4%); decreased appetite (29.6%)
	Taylor et al. (2020) (55)	Phase IB/II evaluated the effect of Pembrolizumab plus lenvatinib in 30 patients with aRCC after failing previous therapies.	- ORR was 70%.	ɸ Hypothyroidism (42%), adrenal insufficiency (7%), hypothyroidism (6%), colitis (4%), thyroiditis, autoimmune thyroiditis (4%)
Ipilimumab	Hammers et al. (2017) (50)	Phase I (CheckMate 016) study evaluated the combination of ipilimumab plus nivolumab in 194 patients. 2 groups of patients were analysed: N3I1 (NIV 3mg/kg plus IPI 1mg/kg) and N1I3 (NIV 1mg/kg plus IPI 3mg/kg).	- ORR was 40.4% N3I1 and N1I3 groups; - Median OS was not reached (N3I1) versus 32.6 months (N1I3); - Grade 3/4 AE occurred in 38% (N3I1) versus 61.7% (N1I3).	N3I1: fatigue (51.1%); rash (31.9%); pruritus (31.9%); nausea (27.7%); arthralgia (25.5%). N1I3: fatigue (68.1%); nausea (44.7%); diarrhea (44.7%); pruritus (36.2%); increased lipase (34.0%).
	Tomita et al. (2020) (51)	Phase III study (CheckMate 214 with extended follow-up), compared nivolumab plus ipilimumab versus Sunitinib in 1096 naïve patients.	-ORR was 39% (NIV+IPI) versus 31% (SUN);-Median OS was not reached (NIV+IPI) versus 33.4 months (SUN); -Grade 3/4 AE occurred in 58% (NIV+IPI) versus 91% (SUN).	NIV+IPI: pruritus (26%); increased lipase (21%); pyrexia (16%); rash (16%), diarrhoea (13%); SUN: decreased platelets (85%); decreased white blood cells (68%); PPE syndrome (68%) decreased appetite (44%); decreased neutrophils (44%).

**AE** = adverse events; **aRCC** = advanced renal cell carcinoma; **ATE** = atezolizumab; **BEV** = bevacizumab; **EVE** = everolimus; **IFN-α** = interferon alpha; **IL-2** - Interleukin-2;**ITT** = intention to treat; **NIV** = nivulomab; **ORR** = objective rate response; **OS** = overall survival; **PAZ** = pazopanibe; **PD-L1** = Programmed death-ligand 1; **PFS** = progression-free survival; **PPE** = palmar-plantar erythrodysthesia; **SUN** = sunitinib

* Only grade 1-2 AE percentages; **ɸ** Total AE percentages for the set of cancers analysed in the study, among which is aRCC.

### Interleukin-2

IL-2, approved by the Food and Drug Administration (FDA) for metastatic kidney cancer and for metastatic melanoma (3, 11) acts by stimulating the proliferation of T cells, cytotoxic T lymphocytes (CTL) specific to tumours, NK cells and possibly intratumor lymphocytes (2). These immunological effects occur through binding IL-2 to its receptors (IL-2R). IL-2R have subunits α, β and γ, and can be dimeric (IL-2Rβ + IL-2Rγ) or trimeric (IL- 2Rα + IL-2Rβ + IL-2Rγ). The association of IL-2Rα (CD25), IL-2Rβ (CD122) and IL-2Rγ (CD132) subunits result in the trimeric IL-2Rαβγ, which has a high affinity for IL-2. In this association, the main function of CD25 is to increase affinity for IL-2, while CD122 and CD132 (mostly expressed in NK, monocytes, macrophages and CD4+ and CD8+ cells) mediate signal transduction. CD25 is extremely important for the proliferation of immunosuppressive, regulating T cells. However, in its absence, and by IL-2Rβγ action, NK and CD8+ cells can be stimulated to proliferate and kill cells that respond to IL-2 (14, 15). So, the IL-2 formulations that confer advantage are those that allow binding of IL-2 to CD122 and CD132, but which disfavour the association of IL-2 with CD25 (14, 15). Despite presenting lower toxicity when compared to IFN-α, complete and durable results require administration of high doses of IL-2 (HD IL-2) (3). A randomized study performed to compare the outcomes of HD IL-2 and IL-2 showed a greater objective response rate (ORR) (21% versus 13%), response durability and overall survival (OS) in HD IL-2 arm. HD IL-2 was tested in combination with bevacizumab in a phase II study, and the results are shown in Table-1 (13).

### Vaccines

The main objective of the implementation of vaccines in anticancer therapy is the activation of the immune response against cancer cells, overcoming the tolerance generated by the tumour. However, not all types of cancer are susceptible to this therapy. Vaccines are implemented in slow-progressing immunogenic cancers that contain specific tissue proteins (16). It is believed that the choice of the target antigen is the most important decision for the development of an anticancer vaccine, because other than non-directed vaccines (such as tumour lysate vaccines), the vast majority of vaccines are designed to generate T-cell responses against shared tumour antigens (those expressed in cancer cells and healthy tissue) (17). There are different types of vaccines: DNA, mRNA, peptide and protein, dendritic cell (DC) and tumour cell vaccines (18). Many significant scientific advances have been made during the last decade, regarding cancer vaccines development (19).

### Dendritic Cell Vaccines

**Figure 2:**
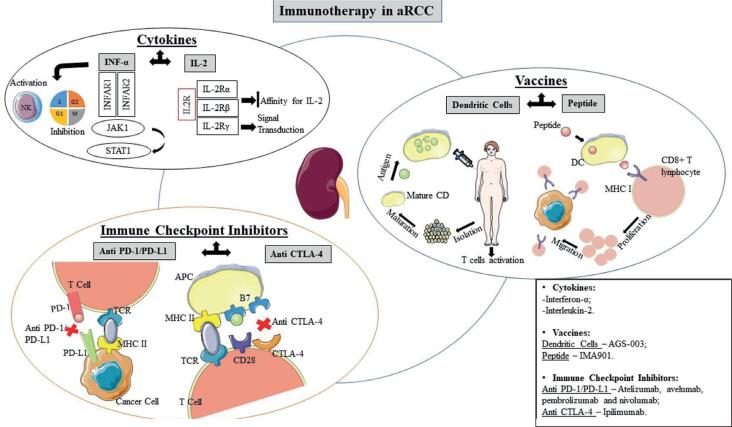
The role of immunotherapy in advanced renal cell carcinoma and its action mechanisms.

Most of the vaccines under development are essentially intended to promote the presentation of tumour-associated antigens by antigen-presenting cells (APC), to generate long lasting immunity through t-cell activation. DC are considered the most effective APC, and for this reason, the effective presentation of tumour antigens by these cells is considered an important factor for the development of cancer vaccines (Figure-2) (19). The first cancer vaccine was sipuleucel-T, a DC vaccine, approved in 2010 by the FDA for the treatment of prostate cancer due to its ability to prolong survival (11, 19, 20).

**APC** - antigen-presenting cells; **CD28** - Cluster of differentiation; **CTLA-4** - cytotoxic T-lymphocyte-associated protein 4; **DC** - dendritic cells; IL - interleukin; IFN-α - Interferon alpha; **IFNAR1** - interferon-α/β receptor 1; **IFNAR2** - interferon-α/β receptor 2; **JAK1** - Janus kinase 1; **MHC I** - major histocompatibility complex I; **MHC II**- major histocompatibility complex II; **PD-1** - programmed cell death-1; **PD-L1** - programmed cell death- Ligand 1; **STAT1**- Signal transducer and activator of transcription 1; **TCR** - T cell receptor.

The immune system can recognize and destroy cells with neoplastic alterations under normal conditions. This mechanism acts as the main defence against cancer cells, and CD8+ T cells are mainly implicated in the process. T cells need tumour antigen presentation made by APC, to stimulate naïve T cell proliferation and differentiation into effector cells. After the recognition of the main antigen complexes class I of the histocompatibility complex (MHC) on the surface of the tumour cell, the T cell tackles cancer cells through different mechanisms, and a subset of specific T cells for the antigen differentiates into memory cells for long-term antitumor protection. DC also contribute to the activation of T helper cells (CD4+), which are also essential to activate CD8+ T cells (19).

One of the known strategies for DC vaccines development involves the use of the patient’s own cells. These cells are first subject to an ex vivo maturation process using toll like receptors (TLR) and agonist cytokines, and then the specific antigens or specific tumour proteins of the patient are loaded. After this process, the cells are injected into the patient in combination with adjuvants, intradermally (11, 19).

### AGS-003

AGS-003 is an immunotherapeutic DC vaccine tested for the treatment of aRCC in combination with sunitinib in a phase II (Table-1) and phase III (discontinued) studies. It is made up of autologous dendritic blood cells, generated by tumour-derived RNA electroporation and CD40 ligand in host immune cells (13, 21, 22). In aRCC, local and systemic effects generated by the tumour, lead to the production of CD40+ cells. However, DC dysfunction hinders the presentation of antigens and consequently the expected response. The administration of AGS-003 helps to circumvent tumour caused effects by presenting mature DC loaded with RNA to produce a more effective and potent response (13).

### Peptide Vaccines

Vaccines based on peptides use protein fragments specifically expressed in tumour cells (18). Peptide vaccines are chemically synthesized, and can be composed by 20-30 amino acids targeting a specific epitope of antigens (18, 23). Despite peptides not having negatively charged backbones, such as DNA and mRNA, the use of delivery vehicles is indispensable to maintain stability, ensure direction and minimize undesirable effects (18).

### IMA901

IMA901 vaccine consists of 9 different human lymphocyte antigens (HLA) class I binding-tumour-associated peptides and one HLA class II binding-tumour-associated peptide (24). Because IMA901 has 10 different peptides linked to the tumour, it promotes an expansion of multiple T cells with different antigen specificities. Induction of CD4+ and CD8+ T-cell responses against tumour-associated antigens causes a broad immune response, although specific against targets functionally relevant to cancer cells. Targeted genes by peptides contained in IMA901 are chosen considering their overexpression in kidney tumour cells, when compared to normal cells (24). IMA901 showed a favourably median OS compared to that obtained in studies with Sunitinib and Sorafenib in a phase II study (25), and a phase III study results (Imprint) are presented in Table-1.

### Monoclonal antibodies

MAb are laboratory manufactured structures (9, 26) to serve as substitute antibodies. They can enhance, restore, or mimic immune system’s action. MAb are formed by two heavy and two light polypeptide chains, bonded by a disulphide bond, resulting in the formation of a “y” structure. This structure includes the variable region (FAB), responsible for recognizing specific antigens and the constant region (FC), responsible for binding the antibody to the cells involved in the immune response. Depending on the antigen, the antibody may generate an antibody-dependent cell cytotoxicity or a complement-system cytotoxicity. These responses can generate inhibition of intracellular signals and membrane receptors blockage (27).

Recently, MAb specifically directed to checkpoints between cancer cells and immune system cells, such as cytotoxic T lymphocyte associated protein 4 (CTLA-4), programmed cell death protein and its binding (PD-1/PD-L1) and adoptive T-cell therapy with Chimeric Antigen Receptor T cell receptor (CAR-T) cells, have shown significant clinical benefit in different types of cancer (28).

### Checkpoint Inhibitors

It is known that cancer cells have few antigens “foreign” to the body because they are derived from their own cells. Although cancer cells are immunogenic, the immunological response can be inhibited by factors contained in the tumour (10).

The adaptive immune response initiates recognizing the antigen by the T cell receptor, with the aid of an APC. This corresponds to the first signal, but to induce cell death, cytokine secretion and memory T-cell formation, a second signal is needed (10, 29, 30). These events, particularly the amplitude and quality of the response are regulated by the second signal, which is given by the inhibitory and/or excitatory factors known as checkpoints (responsible for inhibiting the exacerbation of the immune response, which may lead to an autoimmune response) (20, 29, 31). However, in case of a malignant disease, there is a deregulation in these checkpoints expression (29), with an increase in the expression of inhibitory factors that negatively compromise the action of the immune system against cancer.

Immunotherapy based on the regulation of checkpoints has emerged as a promising cancer treatment strategy, showing significant responses to various antigens (29, 32) and proving efficiency in the treatment of melanoma, lung cancer, bladder cancer, kidney cell cancer and others (33). The most revealing checkpoints studies for cancer treatment include CTLA-4 and PD-1/PD-L1 observed in figure-2 (3, 20, 34, 35).

### CTLA-4

CTLA-4 is the first T cells inhibitory regulator to be identified and tested clinically (30) and it inhibits the response of T cells in primary phases of its activation. For the activation of these cells, the binding of CD28 with the ligands B7-1 (CD80) and B7-2 (CD26) generates the second signal. CD80 is a dimer with a relative high affinity and CD26 is a monomer with lower affinity for CD28. CTLA-4 can interact with both ligands with higher affinity than CD28. Interaction with these ligands serves to inhibit T cells response, although the precise mechanisms are not completely understood (36). However, the replacement of CD28 by CTLA-4 on T cell surface occurs later, thus inactivating its proliferation and function (6, 37). Ipilimumab was the first anti-CTLA-4 to be studied and used in cancer treatment. In aRCC, the combination of ipilimumab plus nivolumab was tested, and results from phase I and III studies are shown in Table-1. Tremelimumab is also a CTLA-4 inhibitor, although less significant since data indicates that its advantages are not superior to that of standard chemotherapy (31).

### PD-1/PD-L1

PD-1 is a molecule expressed on the surface of T cells that binds to its ligand (PD-L1), found in APC. This interaction between the two molecules regulates the induction and maintains the peripheral pathway (31, 38). After initial T cell activation, interactions between PD-1/PD-L1 causes inhibition of its proliferation and cytokines production (Figure-2). Cytoplasmic PD-1 presents a sequence of amino acids involved at the onset of signal transmission; tyrosine is one of these amino acids. When immunoreceptor tyrosine-bases inhibitory motif (ITIM) tyrosine is replaced by phenylalanine, the inhibitory effect generated by PD-1 remains. When immunoreceptor tyrosine-based switch motif (ITSM) tyrosine is replaced by phenylalanine, the inhibitory effect is lost. Therefore, tyrosine in the ITSM region causes the inhibitory effect of PD-1, through recruitment of SHP1 and SHP2. SHP2 in B cells prevents the mobilization of Ca ions and the phosphorylation of IgB, SyK, PLCγ2, ERK1 and ERK2. During T cell activity, PD-1 is accumulated near to T cell receptor (TCR), and SHP2 is recruited to the cytosolic domain of PD-1, where it promotes the dephosphorylation of the molecules responsible for TCR signalling (38). The PD-1/PD-L1 pathway also blocks the phosphorylation of ZAP70 and the function of leukocyte-specific tyrosine kinase, leading to inhibition of TCR signalling (38). Atezolizumab, avelumab, pembrolizumab and nivolumab (first monoclonal antibody approved for the treatment of aRCC by the FDA in 2015) are PD-1/PD-L1 inhibitors tested in aRCC (13, 21), and the results of its clinical trials are presented in Table-1.

## DISCUSSION

The last couple of years have been of utter importance to systemic treatments available for aRCC: the number of approved drugs increased and, most importantly, drugs with better efficacy (39).

Before the use of currently licensed therapies, the treatment of renal cancer was chemotherapy based, with low ORR of approximately 5% (40). After the chemotherapy failure, investigators started to develop systemic treatment involving the use of immune system (8). Cytokine immunotherapies, such as: IL-2 and IFN-α, were established as the standard care, alone or in combination (4, 13). The combination of IFN-α plus bevacizumab was approved by the FDA, but it is no longer used as a single agent, due to the advantages of vascular endothelial growth factor (VEGF) targeted therapies as first-line (13). IFN-α and IL-2 can be associated with high level of toxicity (41) but, Curti et al. demonstrated that the development of AE was significantly associated with improved response and tumour control (42).

Although no better results have been provided with the combination of sorafenib and IFN-α (43) and the combination of bevacizumab with IL-2 plus IFN-α (44), this last association combined with sorafenib improved results (55). This proves that potential benefits can arise from the use of cytokines along with other therapies.

Although cancer vaccines improved outcomes, and showed high safety profile (extremely important, because most approved therapies have serious AE such as cutaneous, gastrointestinal and vascular events) (22), they have failed to demonstrate efficacy in phase III studies, despite evidence of immunological activity. Preclinical data show that cancer vaccines have their greatest effect in settings of low or absent tumour volume, suggesting that the success probability as monotherapy would be increased in prophylactic treatment, reducing the incidence of disease (17, 33). It is believed that the ex vivo preparation of vaccines may change the functionalities and viability of them, in addition to inefficient delivery, because it is possible that administrated vaccines may not be able to reach their targets with precision (11, 18, 19). The other suggested reason may be related to the antigen choice and the immunosuppressive nature of the tumour’s microenvironment, because neoantigens specific T cells are not subject to an optimal microenvironment. Thus, it is possible that combination of vaccines with other therapies (especially those aimed to the microenvironment), may be an option for improving their effectiveness (17). Amin et al. demonstrated that when the AGS-003 was added to sunitinib (first-line treatment for favourable risk), in patients with aRCC with low and intermediate risk, the expected survival was doubled and this combination also presented a good safety profile (22). Curiously, Rini et al. concluded that the combination of sunitinib with IMA901 did not improve relevant outcomes when compared to sunitinib monotherapy (24). The difference between the results might be related to vaccines mechanism of action, since AGS-003 consists of a reinforcement of APC, which helps to stimulate T cells, and IMA901 consists of small fragments of peptides expressed in tumour cells. The contribution of IMA901 becomes ineffective when there is no reinforcement of the APC to help present these antigens. Therefore, the advantages of IMA901 might be clearly expressed in the prevention of recurrences.

In recent years, studies have been developed with more specific immunological agents, which have revolutionized the oncology principles in RCC. The FDA has approved six antibodies that target the PD-1/PD-L1 pathway: atezolizumab, durvalumab and avelumab targeting PD-L1, and cemiplimab, nivolumab and pembrolizumab targeting PD-1 (8).

The Checkmate 025 study showed a significant improvement in the average OS and demonstrated a favourable safety profile (45), which led to an approval of nivolumab in 2015 by the FDA, and in 2016 by the European Medicines Agency (EMA), for patients with aRCC treated with anti-angiogenic agents (46). Stukalin et al. conducted a study that explored the real-world efficacy of nivolumab compared to cabozantinib in the second line setting, concluding that the efficacy was similar for both therapies. This leads to a scenario in which the choice of the therapy to be used as second-line depends more on pragmatic factors, such as: safety profile, availability, price and patient choice (which can be conditioned by the drug’s administration that is intravenous, for nivolumab, and oral, for cabozantinib) (47).

A retrospective study conducted by Kimura et al. concluded that there are possibly no differences in the priority of nivolumab or axitinib as second-line treatment, however, they suggest that, comparing to axitinib, nivolumab should be the choice in aRCC patients with comorbidities (48).

The Checkmate016 and Checkmate214 studies showed that the combination of nivolumab and ipilimumab has a manageable safety profile, durable response and higher efficacy when compared with nivolumab monotherapy and sunitinib, respectively (49-51). This combination therapy is recommended to aRCC patients with clear cell pathology and International Metastatic RCC Database Consortium (IMCD) poor/intermediate risk; patients with clear and non-clear cell pathology with sarcomatoid component (52).

Studies conducted with the combination of pembrolizumab plus axitinib and pembrolizumab plus lenvatinib showed improved outcomes and a manageable safety profile (53-55). The combination of pembrolizumab plus axitinib was shown to induce longer OS tolerable in treatment-naïve patients, compared to first-line sunitinib (52, 56). The results recommend this combination as the present first-line therapy to patients with clear cell pathology with IMCD favourable, poor/intermediate risk and patients with clear and non-clear cell pathology with sarcomatoid component (52).

The Javelin Renal 101 study demonstrated that the combination of avelumab plus axitinib can present an antitumor activity and a manageable safety as first-line treatment, and the study conducted by Vaishampayan et al. also showed greater results in the use of avelumab as first-line treatment (57, 58). Subsequently, Javelin Renal 101 confirmed the efficacy and safety of the combination of avelumab plus axitinib, when compared with sunitinib monotherapy, in terms of PFS while the data were still immature for OS - which is the main reason why this combination is not contemplated on the last guidelines (59, 60).

IMmotion 151 demonstrated a favourable safety profile with the combination (avelumab plus axitinib) over sunitinib, but once again the data were immature to conclude a benefit in OS (61, 62).

Some studies on the use of immune checkpoint inhibitors (ICI) have shown better results in populations with the PD-L1+, however, this does not make the expression of PD-L1 an effective biomarker for predicting the response to anti PD-1/PD-L1 pathway. Therefore, studies demonstrated that the expression of PD-L1 may be associated with both poor prognosis and better responses to therapy. One of the theories attempt to explain this condition, defends that PD-L1 is a dynamic marker that can be regulated by cytokines induced by local inflammation, thus the expression of PD-L1 within the tumour can change over time and according to the microenvironment conditions (59). Other biomarkers, such as the level of total cholesterol (TC) and the expression of sodium-dependent glucose transporter 2 (SGLT-2), have been studied in non-immunological therapies (63, 64). Future studies might focus on the validation of these biomarkers in immunotherapy.

The AE profile is also a condition with great impact on choosing a treatment to be used and it has also impact in the quality of patients’ lives. The results show that the AE profile is similar between drugs from the same family, and in combinations, AE of both classes were observed. Although studies have shown a lower percentage pertaining to the occurrence of AE with ICI when compared to targeted therapy and conventional chemotherapy, ICI has a toxicity spectrum often associated with the immune system (irAE) (38, 46). Several studies showed a relation between these therapies and the occurrence of auto-immune events (54, 55, 57, 65). IrAE may include endocrine, dermatologic, gastrointestinal, hepatic, and other inflammatory events. Regarding PD-1/PD-L1 inhibitors, dermatologic toxicity is the most reported and diarrhoea and colitis may be the most clinically relevant irAE in CTLA-4 inhibitors therapy, which have also led to death (38).

Vaishampayan et al. reported that the most commonly irAE were thyroid disorders (16.1%) and immune-related rash (14.4%) (57). De Giorgi et al. conclude, in a study focused on analysing the safety and efficacy of nivolumab, that in all the AE cases, 50% were considered irAE (diarrhoea, hyperglycaemia, pneumonitis, asthenia, hypertension, skin toxicity, tremor, eyelid ptosis, liver toxicity and hypothyroidism) (65). Studies with pembrolizumab presented colitis, thyroiditis, hypothyroidism, adrenal insufficiency and hyperthyroidism as the most reported irAE (54, 55).

Interestingly, in some cases, the occurrence of AE was associated to better outcomes. Although the reasons for this association are not clearly known, some hypotheses were postulated. It is believed that ICI can cause an immune system unbalance by their cross-reactivity with neoantigens and normal tissue antigens. Another theory defends that increased efficacy in patients with AE may be associated to the interaction between immunotherapy and polymorphisms in genes associated with ICI response. Since PD-1/PD-L1 inhibitors are implicated in the regulation of humoral immunity and influence the production of B cells, altered antibody production may also develop AE (46).

## CONCLUSIONS

The recent years have been critical for the treatment of aRCC. A recent class of drugs, the ICI, showed advantages, with a greater OS, also providing an acceptable quality of life. This class of drugs is already the preconized first-line therapy, in combination with the previously used tyrosine kinase inhibitors or combining two different ICI drugs. The benefits of having a combined therapy are consequent dose reduction and, the reduction of irAE, with the capability to act in different pathways, increasing the treatment efficacy. While some combination regimens wait for mature results, the use of the current first-line therapies as the comparator in the trials will be mandatory and will certainly help us discover new therapeutic options for aRCC cancer patients.

Certainly, immunotherapy has greatly improved treatment of patients with aRCC, however, future studies should, in addition to effectiveness, also focus on ways to reduce toxicity.

## ABBREVIATIONS

AE: = adverse events
APC: = antigen-presenting cells
aRCC: = advanced renal cell carcinoma
ATE: = atezolizumab
AVE: = avelumab
AXI: = axitinib
BEV: = bevacizumab
CcRCC: = clear cell renal cell carcinoma
CTL: = cytotoxic T lymphocytes
CTLA-4: = cytotoxic T lymphocyte associated protein 4
DC: = dendritic cells
EVE: = everolimus
FAB: = variable region
FC: = constant region
FDA: = Food and Drug Administration
GM-CSF: = granulocyte-macrophage colony-stimulating factor
HD IL-2: = high doses of IL-2
HLA: = human lymphocyte antigens
ICI: = immune checkpoint inhibitors
IFN-α: = interferon alpha
IL-2: = interleukin-2
IMCD: = International Metastatic RCC Database Consortium
IPI: = ipilimumab
ISTM: = immunoreceptor tyrosine-bases switch motif
ITIM: = immunoreceptor tyrosine-bases inhibitory motif
ITT: = intention to treat
Mab: = monoclonal antibodies
Mesh: = medical subject headings
MHC: = major histocompatibility complex
NIV: = nivolumab
NK: = natural killer
ORR: = objective rate response
OS: = overall survival
PAZ: = pazopanib
PD-1: = programmed cell death protein
PD-L1: = programmed death-ligand 1
PEM: = pembrolizumab
PFS: = progression-free survival
PPE: = palmar-plantar erythrodysthesia
RCC: = renal cell carcinoma
SUN: = sunitinib
TCR: = T cell receptor
TLR: = toll like receptors
VHL: = Von Hippel-Lindau
